# Large‐Scale FMO‐MP2 Calculations of the Spike Protein Droplet Model

**DOI:** 10.1002/jcc.70052

**Published:** 2025-02-02

**Authors:** Hideo Doi, Tatsuya Nakano, Kota Sakakura, Kazuki Akisawa, Koji Okuwaki, Yoshinori Hirano, Eiji Yamamoto, Kenji Yasuoka, Satoshi Ohshima, Takahiro Katagiri, Yuji Mochizuki

**Affiliations:** ^1^ Department of Chemistry and Research Center for Smart Molecules, Faculty of Science Rikkyo University Tokyo Japan; ^2^ Department of HPC Support Research Organization for Information Science and Technology Kobe Japan; ^3^ Foundation for Computational Science Kobe Japan; ^4^ JSOL Corp., KUDAN‐KAIKAN TERRACE Tokyo Japan; ^5^ Department of Mechanical Engineering Keio University Yokohama Kanagawa Japan; ^6^ Department of System Design Engineering Keio University Yokohama Kanagawa Japan; ^7^ Research Institute for Information Technology, Kyushu University Fukuoka Japan; ^8^ Information Technology Center, Nagoya University Nagoya Aichi Japan; ^9^ Institute of Industrial Science, the University of Tokyo Tokyo Japan

**Keywords:** droplet model, FMO, fragment molecular orbital method, MP2, second‐order Møller‐Plesset perturbation, spike protein, supercomputer Fugaku

## Abstract

The spike protein of SARS‐CoV‐2 is a challenging target for theoretical approaches. Here we report a benchmark calculation of the spike protein droplet model by the fragment molecular orbital (FMO) at the second‐order Møller‐Plesset perturbation (MP2) level on the supercomputer Fugaku. One hundred structure samples from molecular dynamics (MD) simulations were used for both the closed and open forms of this protein (PDB IDs 6XLU and 6XM0 respectively). The number of total fragments is about 20,000, and the job time per structure was about 2 h on 8 racks of Fugaku.

## Graphical abstract

The FMO‐MP2/6‐31G* calculation has been applied to a series of droplet models of spike proteins (PDB ID 6XLU and 6XM0), with a job time per structure of about 2 h using 8 racks (3072 nodes) of the supercomputer Fugaku.

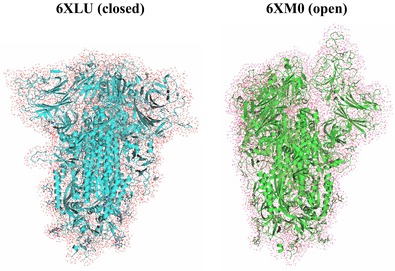

The spike protein of the new coronavirus (SARS‐CoV‐2) (which consists of 3 chains with about 1.1 thousand amino acid residues) plays a key role in the infection of human cells, and the ability to infect varies depending on the mutations, so research in this area is still vigorously pursued [1, 2, 3, 4]. It should be noted here that the receptor binding domain (RBD) is critical for the binding of angiotensin converting enzyme 2 (ACE2) in the early stages of infection. In theoretical studies of spike proteins, molecular dynamics (MD) simulations using classical force fields (FF) are becoming increasingly common. In contrast, there are few examples of studies dealing with electronic structure theories. There are three density functional theory (DFT)‐based reports [5, 6, 7] that focus on RBD. The fragment molecular orbital (FMO), which is applicable to large biomolecules such as proteins, was originated by Kitaura [8] and has been widely used in biophysics and drug design [9, 10, 11, 12] because the derived inter‐fragment interaction energy (IFIE) [13] or pair interaction energy (PIE) [14] is useful for the purpose of analyzing interactions in target systems. At the second‐order Møller‐Plesset perturbation (MP2) level [15, 16], several RBD complexes with ACE2 and Fab antibody were studied in detail using FMO interaction analyses [17, 18, 19, 20]. On the other hand, the third‐order perturbation treatment [15, 16] (FMO‐MP3) [21] was applied to two whole spike proteins (PDB IDs [22] 6VXX of RBD‐closed form and 6VYB of RBD‐open form) [23] as well as several RBD (covering variants) complexes [24], using the computational resources of the supercomputer Fugaku. Note that a different approach with the technique of fragmentation was recently applied to the 6VXX structure [25].

The information obtained from the electronic state calculations above‐mentioned (DFT or FMO) is certainly valuable (especially for residue‐specific interaction analysis), but it must be pointed out that the analyses are based on static single structures and do not take into account the fluctuation effect in the real body situation. Recently, based on the enhanced power of computers, FMO calculations have been performed on droplet‐like structural models generated by MD simulations, and statistical interaction analysis with numerical indices (such as averages and standard deviations) has become practical [26, 27, 28, 29, 30, 31, 32]. In fact, Refs. [28, 29]. report analyses of the main protease (Mpro) of SARS‐CoV‐2 and demonstrate the need to account for structural fluctuations. Besides the omission of the structural fluctuation, the FMO calculation for the whole spike protein of Ref. [23] had two problems, the neglect of hydration and the removal of the sugar moiety (despite the glycoprotein), due to the limitations of the ABINIT‐MP program [12, 33] used at that time. Note here that the droplet model for proteins is likely to be practically superior to the Poisson‐Boltzmann (PB) model under FMO [34, 35] for example in terms of execution speed.

In this short communication, we report a series of calculations on the spike protein droplet models with the ABINIT‐MP program, which has been improved in recent years to speed up FMO‐MP2 calculations [36, 37, 38] on Fugaku‐type (or Fujitsu A64FX) supercomputers and also to support large systems with more than 10,000 fragments; the latest official release version is Ver. 2 Rev. 8 on August 2023 [39, 40]. The droplet structures (a total of 101 samples for each closed and open form) were derived from the trajectories of MD simulations and several attempts were made to handle ions. Surface sugar moieties linked to Gln were included. The computational scheme is as follows.

For both closed and open forms of the spike protein, alternative PDB structures (6XLU and 6XM0 [41], respectively) were selected; there are several missing parts in 6VXX and 6VYB [22], and which were then compensated by the homology modeling using the MOE tool [42] at the structure preparation stage of Ref. [23]. The total number of amino acid residues and sugar moieties was 3363 and 63, respectively. Figure 1 shows the graphical images of these two structures depicted by the PyMOL system [43]. It can be seen that the RBD of chain B (sky color) is directed upwards in the 6XM0 structure of open form. For these native PDB structures, standard operations such as hydrogen addition were performed with MOE [42] as was done in Ref. [23].

**FIGURE 1:**
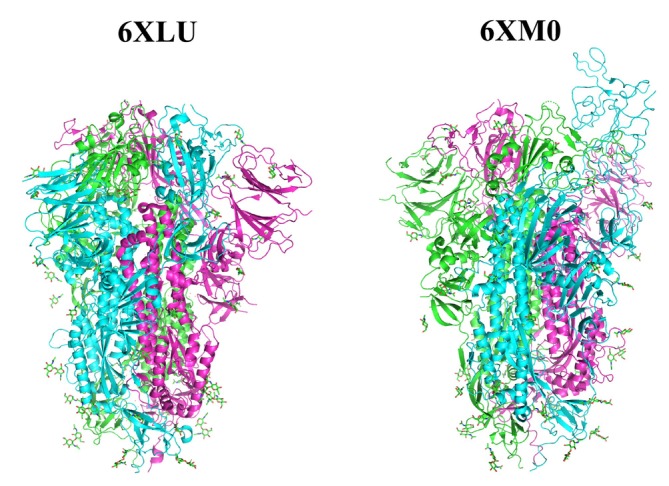
Graphical image of the spike protein of PDB‐IDs 6XLU and 6XM0 (by using PyMOL). The former is of closed form and the latter is of open form due to RBD. The colors green, sky and purple correspond to chains A, B and C, respectively. The sugar moieties are visible on the surface.

The GROMACS program [44] was used (on the supercomputer Fugaku) to perform the MD simulations for these spike proteins, with the Amber14sb [45] set as FF. The settings of the MD were a time step of 2 fs, a temperature of 310 K and a pressure of 0.1 Mpa, and all hydration and ionic Na and Cl conditions were imposed (concentration of 0.15 M in the simulation box with neutral charge). In the 200 ns trajectory obtained by MD, the structures were considered to have reached equilibrium after 100 ns. A total of 101 droplet structures (1 ns time interval) were then generated from the trajectory data, with water molecules more than 4 Å away from the protein deleted and ions retained [28, 32]. The number of remaining water molecules in the droplet model ranged from 12,674 (13,521) to 13,673 (14,376) for 6XLU (6XM0). The total number of ions for 6XLU and 6XM0 was 1558 and 2334, respectively. Overall, the total number of fragments (before the clustering described as below) in the droplet model was about 20,000; there were more fragments for the 6XM0 droplet. Note that the total number of atoms was about 100,000.

In the present calculations, several Python scripts [39] were used as pre‐processors for the droplet model structure file in PDB format. There are three functions as follows. (I) The effective total number of fragments can be reduced by clustering 4 or 5 water molecules that are far from the protein and therefore do not have direct interactions; the water molecules near the protein were treated as individual fragments. (II) Information tables (to be read in by ABINIT‐MP) associated with the fragmentation are generated, even supporting the bond between Gln and the sugar moiety. (III) The information (formal charge and position) of the ions is extracted and written to a separate file, which is read by a locally modified ABINIT‐MP Version 2 Revision 8. The contribution of the ions as classical point charges due to (III) is then calculated according to the integral expression for the nuclear charge [46] in the Hartree‐Fock (HF) step of both monomer and dimer stages.

After the pre‐processing, the effective number of fragments in the droplet model was about 10,000 (or halved). The FMO‐MP2 calculation (with the frozen‐core restriction) was performed for each droplet structure, using the 6‐31G* basis set [47, 48] as the standard choice in recent FMO studies [10]. Forty‐eight OpenMP threads were used for intra‐fragment processing on a single node (14 GB of memory setting), and 3072 MPI processes were invoked for the list of fragments (monomer and dimer) on 8 racks (384 nodes per rack) of the supercomputer Fugaku. Namely, a dual level parallelism of FMO [21, 33, 38] was adapted.

With a small subset of the droplet list, pilot calculations were performed (with a pre‐release internal version of Ver. 2 Rev. 6 from the Fall of 2022 to early Spring of 2023) and found that the dimer HF calculations around the Na(I) ion do not converge frequently; in particular, for 6XM0, only one of the 11 attempts was completed. This is because the distances between the Na(I) ions and the residues (and water molecules) in the classical FF were too close for quantum chemical calculations. Therefore, replacing Na(I) with the smaller Li(I) was tried for production level calculations; the Cl(I) ions were left as they were. Deleting all ions was also tried. Eventually, the classical point charge approximation (due to the above‐mentioned (III)) was adopted with a locally modified version of Ver. 2 Rev. 8 in Spring 2024. A partial renormalization (PR) modification [49] was applied to the MP2 energy to reduce the tendency to overestimate the stabilization energy [23].

Charged amino acids such as Lys and Arg have been known to play an important role in stabilizing the spike protein itself, altering structures, and binding to ACE2 [50, 51, 52, 53, 54]. Based on the IFIE data obtained in Ref. [23], we also showed that the mode of stabilization interactions of the three protein chains differs between the closed form (6VXX) and the open form (6VYB) using tensor decomposition [55]. For these backgrounds, this short communication focuses on the influence of the treatment of ions on the inter‐chain interaction energy (or IFIE sum of residue—residue pairs between chains). The contribution of sugar moieties on the surface of each chain is also considered. A Python script was used for this IFIE summation as post‐processing. Table 1 shows the mean and standard deviation of the energy for each chain combination. The completion rate for the 101 drop structure samples is also shown.

**TABLE 1:** List of inter‐chain interaction energies at the FMO‐MP2/6‐31G* level (average and standard deviation) (in kcal/mol).

		Chain pair	MP2 average	St. dev.	MP2 (PR) average	St. dev.	(Trials)
6XLU	Li(I) ion						(85/101)
		A–B	−1626.9	230.0	−1599.9	230.5	
		A–C	−1981.8	276.9	−1954.4	276.9	
		B–C	−1975.1	185.9	−1947.0	185.7	
	pnt. chg.						(101/101)
		A–B	−1636.1	221.8	−1608.9	222.3	
		A–C	−1972.3	264.0	−1945.2	264.0	
		B–C	−1952.4	207.8	−1924.3	207.6	
	w/o ion						(96/101)
		A–B	−1829.3	180.7	−1802.2	180.6	
		A–C	−2032.7	246.8	−2005.6	246.8	
		B–C	−2029.3	190.4	−2001.2	190.0	
6XM0	pnt. chg.						(100/101)
		A–B	−1583.3	140.3	−1558.0	139.8	
		A–C	−1829.5	213.3	−1804.6	212.9	
		B–C	−1438.0	166.6	−1415.9	166.3	
	w/o ion						(99/101)
		A–B	−1676.5	147.0	−1651.2	146.3	
		A–C	−1892.0	213.5	−1867.1	213.0	
		B–C	−1462.3	169.4	−1440.3	169.1	

*Note:* PR‐modified values are also included. The letters “pnt. chg.” and “w/o ion” mean “point charge replacement” and “without ion” respectively. The job completion rate (against trials) is also shown. For 6XM0, even Li(I) ion replacement did not provide a sufficient number of completed jobs for statistical evaluation, and so the results are not shown.

First, the results of the 6XLU droplet model in Table 1 are discussed. The inter‐chain (A–B, A–C, and B–C) interaction energies are considerable due to the presence of many charged residue pairs with large electrostatic interactions, which can be captured at the FMO‐HF level; the results of the energy decomposition analysis (PIEDA) [14, 56, 57] are given in the Supporting Information (SM). The present values without ion are about 3/4 of the value in Ref. [23], and this is due to the influence of hydration. The PR modification slightly reduces the interaction energy. Interestingly, the standard deviation values indicate that the structural fluctuations of the spike protein are remarkable, suggesting the need for a combined MD‐FMO simulation. It is noteworthy that the values obtained by Li(I) replacement and the point charge approximation are almost the same. In contrast, the interaction energies are overestimated in calculations where the ions are deleted, and it can be seen that such calculations are not desirable. As for the completion rate, the point charge approximation works well, as expected.

For 6XM0, where the RBD portion of chain B is open, the results for the point charge approximation and no ion are shown in Table 1. As shown in Ref. [23], the interaction energies of the A–B and B–C chain pairs are significantly reduced compared to 6XLU because the RBD is on the outside and then the internal stabilization decreases. The importance of considering both the structural fluctuation and the effect of ions (in terms of the point charge approximation) is the same as in the case of 6XLU.

Next, the computational timing is checked. Table 2 summarizes the timing of FMO calculations for the point charge approximation and no ion. Except for the case of 6XLU without ion, it is found that the time required for a job of a single droplet structure is about 2 h (taking into account the standard deviation). The monomer processing takes more than half of the total time because the self‐consistent charge (SCC) condition must be satisfied in the monomer HF stage of the FMO as a Coulomb embedding scheme [8, 9, 10, 11, 12]. The imbalance of charge distributions in the droplet system without ion may affect the HF convergence for 6XLU. It is worth noting that the time required for the Dimer‐ES approximation (dimer processing without HF) is short. In ABINIT‐MP, this step can be performed efficiently without loss of accuracy through continuous multipole expansion [58], allowing even a droplet model with 10,000 fragments to be easily processed; some improvements were made for Dimer‐ES between Rev. 6 and Rev. 8. Another feature of ABINIT‐MP is that the MP2 step can be completed in a short time [39]. The time of Dimer (Total) includes the final processing time for the PIEDA [14, 56, 57] and the time for outputting the long log file, and reducing these parts should be a future target for improvement.

**TABLE 2:** Time data (average and standard deviation) of the FMO‐MP2/6‐31G* job on 8 racks of Fugaku (in seconds).

	6XLU				6XM0			
	pnt. chg.		w/o ion		pnt. chg.		w/o ion	
	Average	St. dev.	Average	St. dev.	Average	St. dev.	Average	St. dev.
Monomer HF	3561.1	736.2	5075.2	903.0	3481.1	694.8	3569.2	643.7
Monomer MP2	15.7	0.3	22.4	1.1	15.7	0.4	16.2	0.2
Monomer (total)	3670.9	736.2	5164.7	904.5	3595.0	694.9	3626.5	643.7
Dimer‐ES	282.1	15.8	663.5	5.9	292.5	14.4	663.6	6.1
Dimer HF	824.1	47.4	1596.6	145.3	855.5	61.7	919.0	63.0
Dimer MP2	350.4	29.8	416.3	44.6	343.7	46.4	358.6	46.0
Dimer (total)	2971.7	46.6	4174.5	152.4	3074.7	55.1	3365.9	79.4
FMO (total)	6642.6	739.3	9339.2	959.3	6669.7	708.4	6992.4	641.1

*Note:* The timing values were taken from the output log files of ABINIT‐MP. The letters “pnt. chg.” and “w/o ion” mean “point charge replacement” and “without ion” respectively. Calculations without ion were done with a pre‐release internal version of Ver. 2 Rev. 6 from Fall 2022 to early Spring 2023, and those with the point charge approximation were done with a locally modified Ver. 2 Rev. 8 in Spring 2024.

In this short communication, we have reported large‐scale FMO‐MP2/6‐31G* calculations for a set of spike protein droplet models (PDB IDs [41] 6XLU and 6XM0) on the supercomputer Fugaku, where the job time per droplet structure was about 2 h using 8 racks or 3072 nodes. Several Python‐based scripts were used for both pre‐processing (such as fragmentation) and post‐processing (statistical evaluation of IFIE). The approach of treating the ions as point charges was extremely effective in improving the job completion rate; this could be a practical technique for FMO calculations of MD‐derived droplet models, not only for the spike protein. It has been shown that the internal inter‐chain interaction energies of the spike protein are adversely affected by the deletion of ions. More detailed biophysical investigations based on the present computational results will be reported elsewhere. Our work with ABINIT‐MP is in progress for upcoming FMO‐MP2 calculations of the spike protein system involving lipid membranes (where the number of fragments after pre‐processing is up to more than 20,000). The Gordon Bell Prize at the Supercomputing 2024 (SC24) conference was awarded for the outstanding achievement of a fragmentation‐based MP2 gradient with GPU acceleration [59]. This award will attract more attention to high‐performance quantum chemistry calculations using fragmentation schemes.

Several improvements of ABINIT‐MP [12, 33, 39, 40] have been underway with the JHPCN subjects jh210036‐NAH, jh220010, jh230001 and jh240001 on the supercomputer Flow (type I) at Nagoya University. Both FMO (by ABINIT‐MP) and MD (by GROMACS) calculations were performed using the HPCI subjects hp220025, hp220352, hp230017 and hp240030 on the supercomputer Fugaku at the Riken Center for Computational Science (R‐CCS). The present work was supported by Rikkyo SFR. Finally, TN and YM would like to thank Prof. Kaori Fukuzawa (Osaka University) for her discussion of ions in the droplet model.

## Supporting information


Data S1.

## Data Availability

The data that support the findings of this study are available from the corresponding author upon reasonable request.
