# Supplementary material for: Large‐Scale FMO‐MP2 Calculations of the Spike Protein Droplet Model
Source: J Comput Chem. 2025 Feb 2;46(4):e70052. doi: 10.1002/jcc.70052 (PMC11788466; doi:10.1002/jcc.70052)
Supplement: Supplementary file 1 — Data S1. [file JCC-46-0-s001.pdf]

Supporting Material (SM)

Large-scale FMO-MP2 calculations of the spike protein droplet model

Hideo Doi[a)], Tatsuya Nakano[b)], Kota Sakakura[c)], Kazuki Akisawa[a)], Koji Okuwaki[a),d)],

Yoshinori Hirano [e)], Eiji Yamamoto[f)], Kenji Yasuoka[e)],

Satoshi Ohshima[g)], Takahiro Katagiri[h)], Yuji Mochizuki[a),i)*]

a) Department of Chemistry and Research Center for Smart Molecules, Faculty of Science, Rikkyo University, 3-34-1 Nishi-ikebukuro, Toshima-ku, Tokyo 171-8, Japan

b) Department of HPC Support, Research Organization for Information Science and Technology, 1-5-2 Minatojima-Minamicho, Chuo-ku, Kobe, 650-0047, Japan

c) Foundation for Computational Science, 7-1-28 Minatojima-Minamicho, Chuo-ku, Kobe, 650-0047, Japan

d) JSOL Corp., KUDAN-KAIKAN TERRACE, 1-6-5 Kudan-minami, Chiyoda-ku, Tokyo 102-0074, Japan

e) Department of Mechanical Engineering, Keio University, 3-14-1 Hiyoshi, Kohoku-ku, Yokohama-shi, Kanagawa 223-8522, Japan

f) Department of System Design Engineering, Keio University, 3-14-1 Hiyoshi, Kohoku-ku, Yokohama-shi, Kanagawa 223-8522, Japan

g) Research Institute for Information Technology, Kyushu University, 744 Motooka, Nishi-ku, Fukuoka-shi, Fukuoka, 819-0395, Japan

h) Information Technology Center, Nagoya University, Furo-cho, Chikusa-ku, Nagoya-shi, Aichi, 464-8601, Japan

i) Institute of Industrial Science, The University of Tokyo, 4-6-1 Komaba, Meguro-ku, Tokyo 153-8505, Japan

*Corresponding author: Yuji Mochizuki (fullmoon@rikkyo.ac.jp)

Pair interaction energy decomposition analysis (PIEDA)[1,2] was actually imposed. The electrostatic (ES), Pauli exclusion (EX) and charge transfer plus other mixing (CT) terms were calculated at the HF level. The so-called dispersion (DI) term was evaluated at the MP2 level with the partial renormalization (PR),[3] but it should include both the pure dispersion contribution and the stabilization due to the correlation-induced reduction of the excess ionicity at the HF level. The former term was therefore calculated separately by the local response dispersion (LRD)[4] with the label "DI(LRD)", based on the recent extension[5] in ABINIT-MP; the rest (correlation) energy is given as "DI(rest)".

Table S1 shows the PIEDA results of inter-chain interaction energies (average and standard deviation), where the sum of all PIEDA terms is equal to the total IFIE (labeled as "MP2(PR)", which already appeared in Table 1). The ES term is certainly of leading stabilization in inter-chain interactions of the spike protein. The effect of ions is reflected in the ES term, as expected. It is interesting to note that the DI(LRD) contribution is rather large compared to the CT term.

References

1. D. G. Fedorov, K. Kitaura, *J. Comput. Chem.* **2007**, *28*, 222.
2. T. Tsukamoto, K. Kato, A. Kato, T. Nakano, Y. Mochizuki, K. Fukuzawa, *J. Comput. Chem. Jpn.* **2015**, *14*, 1 (in Japanese).
3. C. E. Dykstra, E. R. Davidson, *Intern. J. Quant. Chem.* **2000**, *78*, 226.
4. T. Sato, H. Nakai, *J. Chem. Phys.* **2009**, *131*, 224104.
5. S. Matsuoka, K. Sakakura, Y. Akinaga, K. Akisawa, K. Okuwaki, H. Doi, Y. Mochizuki, *J. Comput. Chem.* **2024**, *45*, 898.

Table S1. List of PIEDA of inter-chain interaction energies (in kcal/mol). Both average and standard deviation (in parentheses) are shown. The letters "pnt. chg." and "w/o ion" mean "point charge replacement" and "without ion" respectively.

		Chain pair	ES average	EX average	CT average	DI(LRD) average	DI(rest) average	MP2(PR) average
6XLU	pnt. chg.							
		A-B	-1359.6	798.0	-378.6	-531.7	-137.0	-1608.9
			(232.6)	(70.0)	(26.8)	(32.2)	(12.3)	(222.3)
		A-C	-1724.9	829.3	-383.5	-523.8	-142.4	-1945.2
			(274.8)	(70.8)	(26.3)	(26.9)	(12.2)	(264.0)
		B-C	-1665.0	810.6	-381.9	-548.1	-139.9	-1924.3
			(213.9)	(64.0)	(23.4)	(24.0)	(11.9)	(207.6)
	w/o ion							
		A-B	-1543.1	792.7	-383.3	-530.4	-138.0	-1802.2
			(188.4)	(66.5)	(28.1)	(32.0)	(12.4)	(180.6)
		A-C	-1780.5	828.1	-385.7	-524.3	-143.1	-2005.6
			(258.1)	(71.3)	(26.4)	(26.5)	(12.3)	(246.8)
		B-C	-1734.7	810.0	-386.8	-548.0	-141.8	-2001.2
			(194.4)	(64.7)	(24.2)	(23.9)	(12.4)	(190.0)
6XM0	pnt. chg.							
		A-B	-1349.2	766.2	-356.0	-488.6	-130.3	-1558.0
			(145.0)	(70.3)	(24.6)	(24.2)	(11.4)	(139.8)
		A-C	-1581.4	733.3	-348.4	-483.0	-125.2	-1804.6
			(225.3)	(59.8)	(20.9)	(17.7)	(10.7)	(212.9)
		B-C	-1209.0	615.5	-286.7	-432.7	-103.0	-1415.9
			(173.3)	(52.3)	(18.2)	(16.4)	(8.0)	(166.3)
	w/o ion							
		A-B	-1438.6	765.8	-358.5	-488.8	-131.1	-1651.2
			(151.3)	(70.1)	(25.8)	(24.2)	(11.8)	(146.3)
		A-C	-1639.2	733.3	-351.7	-482.9	-126.6	-1867.1
			(224.3)	(59.1)	(22.2)	(17.8)	(11.4)	(213.0)
		B-C	-1230.3	614.7	-288.4	-432.6	-103.7	-1440.3
			(175.9)	(52.2)	(19.0)	(16.4)	(8.3)	(169.1)
